# A transcriptomic survey of *Migdolus fryanus* (sugarcane rhizome borer) larvae

**DOI:** 10.1371/journal.pone.0173059

**Published:** 2017-03-01

**Authors:** Darlan Gonçalves Nakayama, Célio Dias Santos Júnior, Luciano Takeshi Kishi, Rafael Pedezzi, Adelita Carolina Santiago, Andrea Soares-Costa, Flavio Henrique-Silva

**Affiliations:** 1 Department of Genetics and Evolution, Laboratory of Plant Biotechnology, Federal University of São Carlos, São Carlos, Brazil; 2 Department of Genetics and Evolution, Laboratory of Molecular Biology, Federal University of São Carlos, São Carlos, Brazil; Natural Resources Canada, CANADA

## Abstract

Sugarcane, a major crop grown in the tropical and subtropical areas of the world, is produced mainly for sucrose, which is used as a sweetener or for the production of bioethanol. Among the numerous pests that significantly affect the yield of sugarcane, the sugarcane rhizome borer (*Migdolus fryanus*, a cerambycidae beetle) is known to cause severe damage to the crops in Brazil. The absence of molecular information about this insect reinforces the need for studies and an effective method to control this pest. In this study, RNA-Seq technology was employed to study different parts of *M*. *fryanus* larvae. The generated data will help in further investigations about the taxonomy, development, and adaptation of this insect. RNA was extracted from six different parts (head, fat body, integument, hindgut, midgut, and foregut) using Trizol methodology. Using Illumina paired-end sequencing technology and the Trinity platform, trimming and *de novo* assembly was performed, resulting in 44,567 contigs longer than 200 nt for a reunion of data from all transcriptomes, with a mean length of 1,095.27 nt. Transcripts were annotated using BLAST against different protein databanks (Uniprot/Swissprot, PFAM, KEEG, SignalP 4.1, Gene Ontology, and CAZY) and were compared for similarity using a Venn diagram. Differential expression patterns were studied for select genes through qPCR and FPKM comprising important protein families (digestive peptidases, glucosyl hydrolases, serine protease inhibitors and otopetrin), which allowed a better understanding of the insect’s digestion, immunity and gravity sensorial mechanisms.

## Introduction

Insects are ancient, diverse, and adaptable organisms capable of surviving in a vast range of environments [1,2]; the coleopterans are the most diverse among the insects. *Migdolus fryanus* (Coleoptera: Cerambycidae) is a holometabolous insect that presents a long life cycle (3–4 years) in four stages: egg, larva, pupa, and adult. Under laboratory conditions the larvae hatch about 15 to 20 days after oviposition. Newly hatched larvae apparently feed on only organic matter, and in the later stages, they nourish up the root system of plants. During the larval stage, which is the largest in the life time of this insect, numerous galleries are formed in the soil that can reach depths of up to 5 m. This behavior has hampered biological studies of this insect [3].

At the time of feeding, the larvae tend to destroy the root system of sugarcane, which results, first, in the drying of senescent leaves and then other parts of the plant. This ultimately impairs the budding of stumps, thus contributing to a sharp decline in sugarcane productivity. Damages caused by this insect in Brazil range between 25 and 30 tons of sugarcane per hectare and often lead to a complete destruction of the crop [4].

So far, no efficient method to control this pest is available. Chemical pesticides cannot give adequate protection because the larvae bore into the plant rhizome. Other strategies including the use of traps containing pheromone to attract male adults and mechanical collection and destruction of infested stumps showed limited benefits [3,4]. Studies to understand the insect biology and strategies for its control are still scarce.

In recent years, the use of next-generation sequencing technologies (NGS) has allowed the study of numerous insect pests [5,6]. Using high-throughput deep RNA sequencing technology, it is possible to identify the genes implicated in the growth, development, survival and phytophagous behaviors of insects. For example, transcriptome analysis of sugarcane giant borer (*Telchin licus licus*) allowed the characterization of digestive peptidases (mainly serine and amino peptidases), enabling an understanding of the insect’s dietary system as well as the activation/deactivation process of Cry toxins [7]. A 454-pyrosequencing transcriptome profile of all developmental stages of cotton boll weevil (*Anthonomus grandis*), the most important cotton pest in Brazil, permitted the identification of several proteins involved in RNA interference mechanism. These sequences are a reliable source for identifying the candidate genes for insect control [8]. Midgut transcriptome of *Anoplophora glabripennis* (Coleoptera, Cerambycidae) allowed the identification of several genes encoding digestive peptidases, enzymes involved in plant cell wall degradation, detoxification, and nutrient extraction [9].

In the present study, we used the RNA-Seq technology to investigate the transcriptomes of six *M*. *fryanus* larval parts (foregut, midgut, hindgut, integument, fat body, and head). An initial characterization of *M*. *fryanus* digestive peptidases was performed. Serine protease inhibitors (serpins) and glycosyl hydrolases were also investigated due to their roles in different biochemical pathways involved in larvae survival and development. Finally, an otopetrin-like sequence was identified and analyzed due to its putative role in insect spatial orientation. These data provide a basis for further taxonomic, developmental, and adaptive studies on this insect and other Coleoptera.

## Material and methods

### Sample collection and larvae dissection

**Fig 1:**
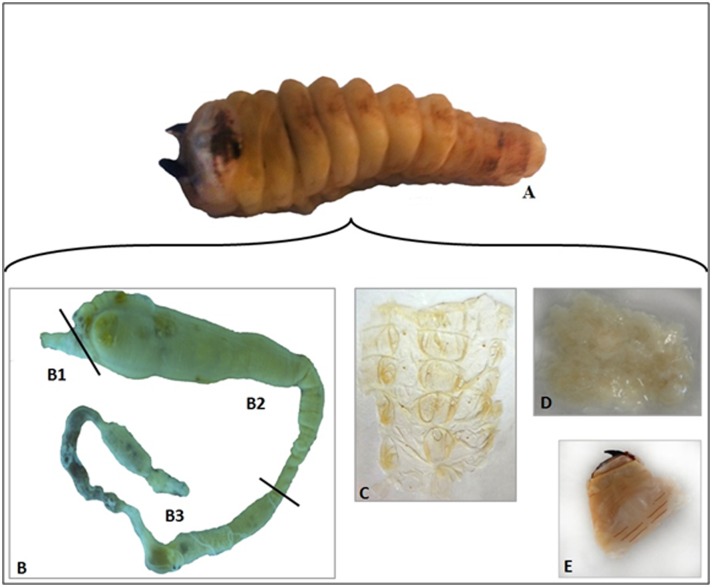
Dissected parts of *M*. *fryanus* larvae body. (A) whole larva. (B) intestinal tube: B1, foregut; B2, midgut; B3, hindgut. (C) integument. (D) fat body. (E) head.

*M*. *fryanus* larvae (Fig 1A) were supplied by Entomol Consultoria Ltda under the responsibility of an agricultural entomologist, Luiz Carlos de Almeida, on July 26, 2013. Larvae were collected directly from field through opening of trenches (50 x 50 x 40 cm depth). We preferred to use native larvae due to the limitation in establish an artificial diet for this insect culture in laboratory. The use of native larvae hampers the estimation of larval age or developmental instar, due to the long life cycle of this insect. However, we procured mature larvae for this study, able to feed on the sugarcane rhizome, with similar size, shape, weight (average length 41 mm and weight 1.95 g), similar head length (average 9.2 mm) and similar head width (average 10 mm).

For rinsing, *M*. *fryanus* larvae surfaces were immersed in sterile phosphate-buffered saline (137 mM NaCl, 2.7 mM KCl, 10 mM Na_2_HPO_4_, 2 mM KH_2_PO4, pH 7,4) and kept on ice for immobilization. Using a magnifier and sterilized scalpels and forceps, parts of the larvae were extracted and kept in the same saline.

Six parts of the *M*. *fryanus* larvae body were selected in order to bring more easy and clear dissection. In addition, they were chosen to comprise all processes of interest (spatial orientation, digestion, immunity, ecdysis, and other functions of metabolism) and almost the whole larval body. The intestinal tube (Fig 1B) was carefully separated into foregut (Fig 1-B1), midgut (Fig 1-B2), and hindgut (Fig 1-B3). Successive washes with sterile phosphate-buffered saline were performed to remove gut contents and microbiota. The integument was dissected from the ventral region of the larvae (Fig 1C). The fat body was carefully removed by scraping the integument (Fig 1D), and the head was separated from other parts (Fig 1E). Pools of head, integument, body fat, hindgut, midgut, and foregut, approximately 100 mg of each tissue, were stored in a freezer at –80°C until RNA extraction.

### RNA extraction

For total RNA extraction, 100 mg of each dissected tissue was mixed with liquid nitrogen and treated with Trizol^®^ reagent (Life Technologies), at a proportion of 5 μL Trizol for each milligram of tissue, following manufacturer’s instructions. Total RNA was quantified using a Nanodrop ND-1000 spectrophotometer (Thermo Scientific), with its integrity checked in 1% agarose gel using 1x Tris-acetate-EDTA buffer (40 mM Tris-acetate, 1 mM EDTA, pH 8.0) and visualized after ethidium bromide staining. Each sample of total RNA was individually treated with RNAse-free DNase I (Invitrogen) to eliminate traces of genomic DNA in reactions containing 3 μg of RNA in a final volume of 20 μL, as described by manufacturer’s instructions.

### cDNA library construction and sequencing

Beads with oligo(dT) were used to isolate poly(A) mRNA from total RNA obtained from each tissue. Subsequently, mRNA was fragmented through its incubation with a Fragmentation Buffer 1x supplied with Illumina Small RNA Sample Preparation Kit. Taking these fragments as templates, SuperScript II (Invitrogen) enzyme and random hexamer-primers were used to synthesize the first-strand cDNA followed by second-strand cDNA with DNA polymerase I and RNase H supplied by Illumina Kit. The fragments were end-repaired, 3′ ends were adenylated, and adapters were ligated. Suitable fragments (200 ± 30 bp) were selected by agarose gel electrophoresis, purified, and enriched by polymerase chain reaction (PCR) with adapter-specific primers. The quality of the libraries was validated using Agilent 2100 Bioanalyzer (Agilent Technologies). Finally, these libraries were sequenced using HiSeq1000 (Illumina) in paired-end reads (2x100 bp), according to the manufacturer’s instructions. Raw data from sequencing runs were submitted to the Sequence Read Archive repository of the National Center for Biotechnology Information under accession number PRJNA259776.

### *De novo* assembly of Illumina reads

Before starting transcriptome assembly, a filtering process of raw sequencing reads was performed to eliminate low-quality reads. We used the NGSQCToolkit_v2.3.3 [10] to remove the paired-end reads containing unknown nucleotides “N” or those with bases having Q<20 exceeding 10%. Clean reads thus obtained were used for *de novo* assembly using the Trinity platform [11]. K-mer size of 25 was used for all analyzed transcriptomes.

### Functional annotation

Transcripts were annotated using BLAST algorithm against the UniProt/Swissprot databank using a Dual Xeon 2.2 GHz 8-core server with 384 GRAM, 15 HD Tera, and E-value cut-off of 10^−5^. The categorization based on Gene Ontology (GO) terms and searching of protein domains (PFAM) were performed using InterproScan 5 program, and enzyme classification was performed using the KEGG Automatic Annotation Server program (http://www.genome.jp/tools/kaas). The prediction of presence and location of signal peptides from protein sequences in all transcriptomes was performed using SignalP 4.1 program. CAZY (Carbohydrate-Active Enzymes Database; http://www.cazy.org) was used as a reference to evaluate a large number of enzymes related to carbohydrate metabolism.

### FPKM analysis

The expression levels of all transcripts identified were calculated as the average fragments per kilobase of exon per million fragments mapped (FPKMs) as described by Trapnell et al. [12]. TopHat and Cufflinks [12] were used to estimate the relative abundance of these transcripts. We used a default FPKM threshold of 1 to consider a transcript expressed. In order to compare with qPCR values, the tissue with a higher FPKM value was used to normalize other values for each tested transcript.

### Venn diagram

A preliminary Blastx was carried out with gut tissues against themselves (foregut, midgut, hindgut) and non-gut parts against themselves (head, integument, fat body). Subsequently, each transcript with a cut-off of identity >97% and E-value <1x10^-5^ was associated as a match. The counting of equal transcripts was performed using a homemade Perl script, and graphs were generated using R package.

### Quantitative Real-Time Polymerase Chain Reaction (qPCR) assays

Fourteen target transcripts were selected (S1 Table) to discuss aspects of their tissue-specific expression patterns and inferences about their possible functions for insect and possible applications. Selected transcripts included potential digestive proteases (cathepsins and serine peptidases), glycosyl hydrolases, serine peptidase inhibitors (serpins) and an otopetrin-like transcript, which could work as a gravity sensor essential for spatial orientation. To the best of our knowledge, this study is the first to identify and further characterize an otopetrin-like transcript in a Cerambycidae member.

Total RNA was isolated from each tissue sample (head, integument, fat body, foregut, midgut, and hindgut) as mentioned earlier. The first-strand cDNA synthesis was performed using 500 ng of total RNA in a 20 μL reaction using ImProm-II^™^ Reverse Transcription System (Promega). qPCR was performed using specific primers to detect the relative levels of serpins, cathepsins, serine peptidases, glycosyl hydrolases, and an otopetrin-like transcript (Table 1). Primers were designed using the Primer 3 program, version 4.0 [13]. qPCR amplification reactions were conducted using 5 μL of cDNA and no-template control (NTC) in three technical replication assays of 10 μL of reaction mixture. Thermal cycling conditions using KAPA SYBR FAST qPCR kit were: enzyme activation 95°C for 3 min, followed by 40 cycles of 95°C for 30 s and 60°C for 40 s. After amplification, melting curves were constructed and data analysis was performed using Eco Real-Time PCR System Support (Illumina). NTC of each replicate was run to detect contamination and determine dimer formation.

**Table 1:** Oligonucleotides used in qPCR experiments.

Name	Sequence 5′→ 3′
Mf_catL_Fw	CCACGGACTTAGATCACGA
Mf_catL_Rv	ATCCATTTTCACCCCAATC
Mf_catB_Fw	GAGGGCTCGTTCGTAAACT
Mf_catB_Rv	TAATTTCCATCCGCATTTG
Mf_sp1_Fw	AATGTCACCCTCCCAACCAT
Mf_sp1_Rv	GGAGGTACCCGCTTCTCAAT
Mf_sp2_Fw	GCGCAGAACTTTCAGAACGA
Mf_sp2_Rv	TGGTGCTGTCCTTCTCGTAG
Mf_sp3_Fw	AACTTGGGCTTTAACCGCAG
Mf_sp3_Rv	TTTGAAACACCGACCACCTG
Mf_S1_Fw	CTTTACCGCCACCTCCTAA
Mf_S1_Rv	TATTCTGTTTGGAGGCCG
Mf_S2_Fw	ATGAGAACGGCCAGGATAC
Mf_S2_Rv	TGATGCAGCCGATAAAATAA
Mf_S3_Fw	AGGGCTGCTTTACCAAGAC
Mf_S3_Rv	ACACCTTGTTCGGTGG
Mf_GH9_Fw	CAGCCAGTTCATGCCCTAAT
Mf_GH9_Rv	TTTGATCCGGGCCACTTA
Mf_GH13_Fw	ACGCAGGTCCACCAAGTTAC
Mf_GH13_Rv	ATTTGAGGCCAACGATGTTC
Mf_GH16_Fw	GGGCAATGGTATCTGAGGTG
Mf_GH16_Rv	CCGTTAACAGCTCCCATGTT
Mf_GH35_Fw	CTCAACAACCGCACTGCTTA
Mf_GH35_Rv	ACAGCTCTTCCATCGGCTTA
Mf_GH116_Fw	GGGCAATGGTATCTGAGGTG
Mf_GH116_Rv	CCGTTAACAGCTCCCATGTT
Mf_otopetrin_Fw	GCTATGCTGGAAGTTGTCGG
Mf_otopetrin_Rv	TTCCAGCGGTGACATCTCTT
Mf_RP18_Fw	AAGAAACCTGGACGTGATGG
Mf_RP18_Rv	TCCTGTAACACGCAAAGCAC

The expression of *M*. *fryanus* ribosomal protein 18 gene (RP18) was used as an internal control. We validated this gene as a reference using qPCR experiments in triplicate at minimum for each tissue. After calculation of Ct values, we used a Friedman test do compare averages (S2 Table). This test was followed by a Dunn’s multiple comparison test to compare RP18 expression between all parts of the *M*. *fryanus* larvae. It proved to be that the effect of statistical variation was not significant (Friedman statistic = 9.667, p > 0.05), and there was no significant difference (p > 0.05) in RP18 expression among tested parts. All statistical analyses were carried out using GraphPad Prism v.6 (La Jolla, www.graphpad.com). Based on this gene, we analyzed the relative differences in the transcript levels using the 2^-ΔΔCt^ method [14]. In order to compare with FPKM values, for each tested transcript, the tissue with higher expression level was used to normalize other values.

### Phylogenetic analysis

The phylogenetic analysis was conducted as described by Brand *et al*. [15]. 32 predicted GH9 sequences from 7 different orders were accessed from NCBI database. The proposed LG+I+G model was used to infer an unrooted maximum likelihood (ML) tree in RaxML. A ML phylogenetic reconstruction protein tree was calculated using RaxML v7.2.7 [16]. Ten independent ML searches were carried out on 10 randomized parsimony trees to find the highest likelihood log valued tree. Subsequently, a bootstrapping procedure was realized with 1,000 replicates. In the end, a final tree was mid-point rooted.

## Results and discussion

### Sequence analysis, *de novo* assembly, and annotation

The obtained transcriptomes were named Mf_head, Mf_integument, Mf_fatbody, Mf_foregut, Mf_midgut and Mf_hindgut according to the tissue origin (Table 2). Combining all transcriptomes (Mf_all), a total of 194,671,657 clean reads were obtained after filtering and adapter removal using NGSQCToolkit_v2.3.3. Over 99% of the clean reads had Q>20 for all transcriptomes. These high-quality clean reads were assembled *de novo* using Trinity platform, resulting in 44,567 contigs longer than 200 bp, with a mean length of 1095.27 bp. Our assembly results suggest that length distribution pattern, mean length of contigs, and N50 values are similar to those of other insects obtained from previous Illumina-based transcriptome studies[9,17], leading us to conclude that our transcriptome sequencing data from *M*. *fryanus* larvae was effectively assembled.

**Table 2:** *M*. *fryanus* larvae transcriptome assembly.

	Mf_head	Mf_integument	Mf_fatbody	Mf_foregut	Mf_midgut	Mf_hindgut	Mf_all
**Number of reads before filtering**	31,812,858	31,212,938	30,607,165	34,564,288	34,043,153	32,669,658	194,910,060
**Number of reads after filtering**	31,771,520	31,172,644	30,575,843	34,530,974	33,991,082	32,629,594	194,671,657
**Number of contigs (bp)**	31,403	9,902	30,352	29,384	25,356	25,844	44,567
**Largest contig (bp)**	24,271	36,447	16,920	13,160	11,999	18,932	28,555
**Shortest contig (bp)**	201	201	201	201	201	201	201
**Average length of contigs (bp)**	1,367.12	730.46	1,170.61	1,111.39	823.67	1,076.28	1,095.27
**Total length of contigs (bp)**	68,890,685	17,449,195	60,479,403	54,048,150	44,846,390	47,816,775	85,966,752
**N50 length (bp)**	2,678	1,578	2,288	2,034	1,737	2,023	2,167
**N70 length (bp)**	1,529	615	1,293	1,200	794	1,150	1,170
**N90 length (bp)**	575	288	471	465	311	437	432
**GC (%)**	37.60	39.21	37.55	36.73	36.96	37.14	36.67
**Q20 (%)**	99.87	99.87	99.9	99.9	99.85	99.88	99.88

Assembled contigs were analyzed against UniProt/Swissprot database for similarities with known sequences having an E-value cut-off of 10^−5^. A comparison with PFAM database was also carried out for identification of possible domains in transcripts, prediction of signal peptides, enzyme classification (EC), and functional categorization with GO, in addition to CAZY analysis (Table 3).

**Table 3:** Transcripts and proteins obtained from *de novo* assembly and annotated based on different protein databanks.

	Transcripts	Proteins	UniProt	PFAM	GO	EC	CAZY	SignalP
**Mf_all**	78,498	53,529	31,242	33,442	40,569	5,340	1,206	4,004
**Mf_head**	43,238	38,106	20,165	26,863	27,571	3,466	754	2,808
**Mf_integument**	28,735	17,924	14,150	13,234	14,171	1,868	335	1,109
**Mf_fatbody**	45,122	35,497	20,161	23,862	27,596	3,865	880	2,576
**Mf_foregut**	43,439	34,581	20,481	22,770	26,262	3,547	858	2,170
**Mf_midgut**	40,285	24,130	17,384	16,972	21,081	2,812	522	1,293
**Mf_hindgut**	41,902	31,338	18,842	21,400	24,394	3,276	750	1,984

**Fig 2:**
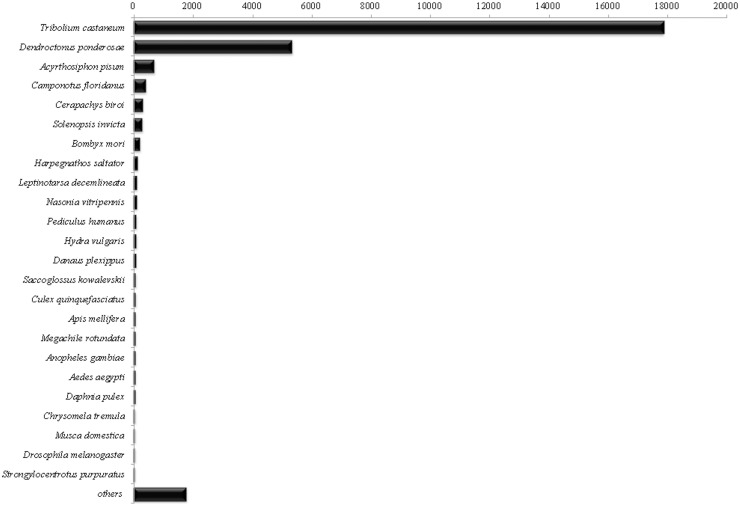
Top BLASTx species distribution against NCBI non-redundant (nr) protein database. A number of contigs matched insect genes, mainly *T*. *castaneum*, another coleopteran. E-value cut-off used was 1x10^-3^.

Similarly, annotation using Blast2GO platform was performed. The BLASTx hits distribution allowed clustering of contigs according to the most frequent species similarities (Fig 2). As was expected, the highest percentage of sequence hits occurred with proteins, particularly Coleoptera (82.51%), Hymenoptera (5.41%), Hemiptera (2.5%), and Diptera (1.21%). A large number of hits was observed for *Tribolium castaneum* (62.78%) and *Dendroctonus ponderosae* (18.82%) from a Blastx search, possibly because a large amount of genomic information is available on these organisms and because they are phylogenetically closer to *M*. *fryanus*.

**Fig 3:**
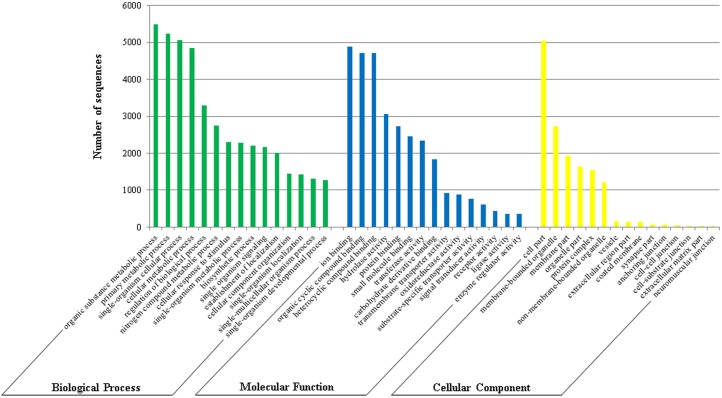
Classification of Mf_all trascriptome based on GO terms “Molecular Function”, “Biological Process,” and “Cellular Component”. The graph indicates an abundance of a specific category of genes in a main category.

GO analysis of Mf_all transcriptome was performed to classify the functions of the predicted proteins. All contigs were classified in level 2 for “Molecular function” (43,060), “Biological process” (31,060), and “Cellular component” (14,792) (Fig 3). “Metabolic” and “cellular” terms were predominant for Biological Process, while the terms “catalytic activity” and “binding” stood out in Molecular Function. For Cellular Component, a major abundance of GO terms for were predicted “cell parts” (5,034). Our data is consistent with other insect transcriptome patterns of GO classification [7].

**Fig 4:**
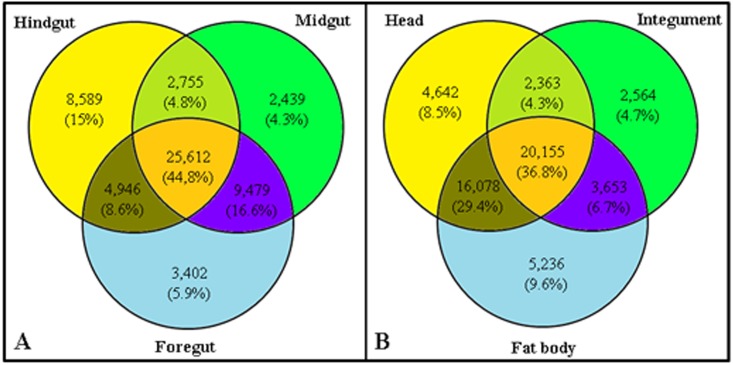
Venn diagrams showing a comparison of *M*. *fryanus* larvae gut transcriptomes (foregut, midgut, and hindgut) and non-gut transcriptomes (head, fat body, and integument). Numbers refer to transcripts that belong to each group. (A) Gut transcriptomes. (B) Non-gut transcriptomes.

A comparative analysis between gut transcriptomes (Fig 4A) and non-gut transcriptomes (Fig 4B) was performed to identify conserved transcripts among these parts of the *M*. *fryanus* larvae body and those that are unique to each tissue. A total of 125,626 gut transcripts were counted, 44.8% of which were shared by all gut tissues. Midgut and hindgut were the most related, with 61.4% transcripts in common.

Among the group of non-gut transcriptomes, with a total of 54,692 transcripts, all three parts shared about 36.8% of common transcripts. Moreover, the most related parts were head and fat body, sharing 29.4% of total transcripts. The tissue with fewest private transcripts was midgut, with only 2,439 transcripts.

About 14 target genes were selected to investigate tissue-specific expression patterns and their possible functions and applications. Selected genes included potential digestive proteases (cathepsins and serine peptidases), glycosyl hydrolases, serine peptidase inhibitors (serpins), and an otopetrin-like transcript, which probably encodes a gravity sensor involved in spatial orientation.

### Digestive peptidases in *M*. *fryanus* transcriptome

**Fig 5:**
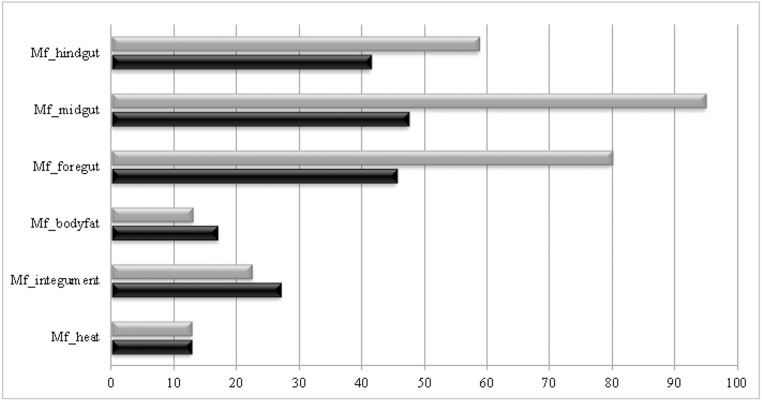
Relative abundance of putative representants of digestive cysteine and serine peptidases sequences in different parts of *M*. *fryanus* larvae. Normalization was accomplished by multiplying the number of sequences obtained for each digestive peptidase by the number of transcripts from each tissue (see Table 3) divided by the total number of transcripts generated (78,498). Gray bars represent serine peptidases. Black bars represent cysteine peptidases.

After annotation against PFAM database, major putative digestive peptidases classes identified were serine and cysteine. Overall, we identified 52 sequences similar to cysteine peptidases of papain family and 217 sequences similar to serine peptidases of chymotrypsin family in all analyzed transcriptomes. To perform a relative abundance analysis, we selected a common ORF representative of each family (cysteine and serine peptidase), which was blasted against all transcriptomes using a cut-off value of 1.0e^-5^. Subsequently, we counted the number of different contigs presenting each protein of this family. These data are shown in Fig 5. These enzymes were abundant in all the transcriptomes analyzed, mainly in gut tissues: midgut > foregut > hindgut. The serine peptidase transcripts were relatively more abundant than cysteine peptidases in the intestinal tract.

Cysteine peptidases are characterized by the presence of a cysteine residue in its active center and its identification is based on its inhibitor specificity (iodoacetate, iodoacetamide and E^-64^). In general, the digestive peptidase activity in coleopterans is related to cysteine peptidases [18,19]. Serine peptidases are proteolytic enzymes whose central catalytic machinery is a “catalytic triad” composed of histidine, aspartate, and a reactive serine. In Lepidoptera and Diptera, serine peptidases are the principal class of digestive enzymes. Cysteine and serine peptidases together are found in few insect groups, including in Thysanura, Hemiptera, and Cucujiformia of the order Coleoptera. In the midgut of *T*. *molitor* larvae, studies showed that serine peptidases had higher activity at alkaline pH (posterior midgut), while cysteine peptidases had higher activity at acidic pH (anterior midgut) [20,21].

### Expression analysis of digestive peptidases from *M*. *fryanus*

**Fig 6:**
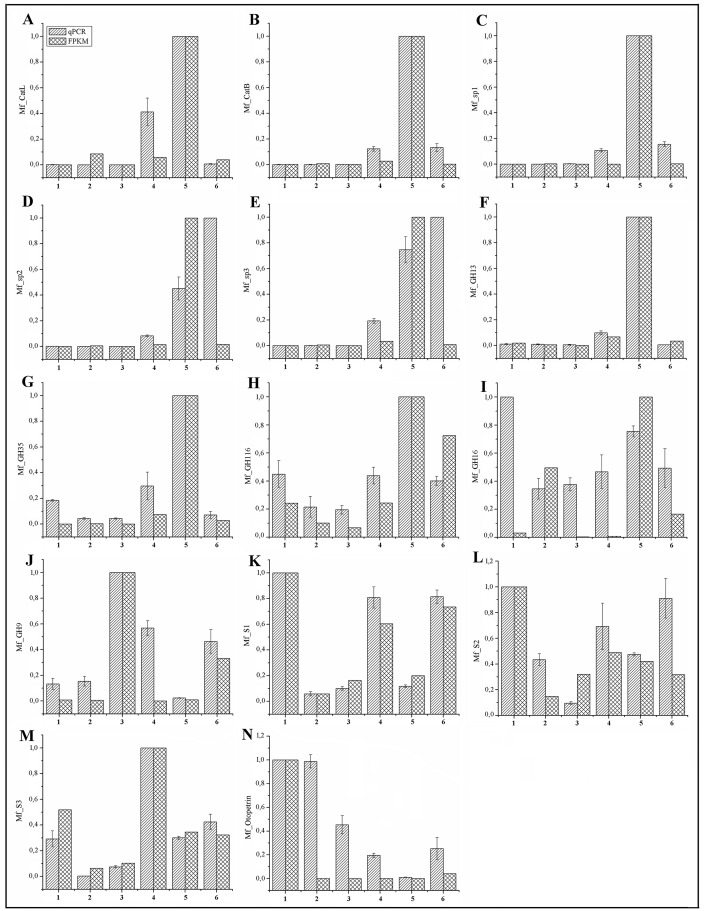
Relative expression of selected genes in different *M*. *fryanus* larval parts by qPCR and FPKM. Each bar represents the relative expression in comparison to a higher expression tissue, arbitrarily designated as 1. Standard errors of technical triplicates are shown. On the x-axis: (1) Fat body. (2) Integument. (3) Head. (4) Foregut. (5) Midgut. (6) Hindgut.

FPKM and qPCR were performed to assess the expression pattern of serine and cysteine peptidases (Fig 6) in fat body, integument, head, foregut, midgut, and hindgut.

We verified the expression levels of cathepsin L (Mf_cathL) (Fig 6A) and cathepsin B (Mf_cathB) (Fig 6B) only in the intestinal tube, being more expressive in the midgut. In the head, integument, and fat body, no expression was observed. Expression values observed by qPCR are in agreement with those observed by FPKM. In insects, cysteine peptidases have a pivotal role in several biological processes. In this case, two cysteine peptidases appear to act in protein digestion. Three serine peptidases (termed as Mf_sp1, Mf_sp2, and Mf_sp3) were further investigated regarding their differential expression (Fig 6). They were supposed to have a digestive function since they showed expression only in gut tissues. The expression of Mf_sp1 (Fig 6C) was higher in the midgut, where it was expressed about 9.32 times higher than in foregut and 6.43 times higher than in hindgut. Mf_sp2 (Fig 6D) showed a higher expression in the hindgut, being 2.21 and 12 times greater than in midgut and foregut, respectively. Mf_sp3 (Fig 6E) also showed a higher expression in the hindgut, being 1.34 and 5.18 times higher than in midgut and foregut, respectively. Although the qPCR data showed differential expression of serine peptidases preferentially in the hindgut, by FPKM analysis the three serine peptidases presented a higher expression in the midgut, as observed for putative cathepsins. More specific studies for determining the pH gradient of *M*. *fryanus* intestinal lumen compartments and its digestive enzymes are necessary for a better understanding of their roles.

In *Sphenophorus levis* (Coleoptera: Cucurlionidae), the sugarcane weevil, protein digestion starts and ends in midgut with a cathepsin L-like proteinase [22], suggesting a divergent pattern from *M*. *fryanus*, where the diversity of digestive proteases could promote an adaptation to a more heterogeneous diet.

Previous studies have demonstrated that cathepsins B, L, and serine peptidases are the major enzymes found in the larval midgut of tenebrionid beetles [23]. The occurrence of cysteine peptidases as digestive enzymes in addition to serine peptidases proved to be an adaptation that allowed the beetles to benefit from foods rich in serine peptidase inhibitors [24]. The high number of serine as of cysteine peptidases observed in our results suggest that *M*. *fryanus* larvae should use both digestive strategies. Studies showed that a lack of cysteine peptidases in some members of Cerambycidae family could be a regress to a digestive strategy based on serine peptidases [25]. *M*. *fryanus* is a polyphagous insect that attacks many economically important crops, including eucalyptus, vine, mulberry, cotton, beans, coffee, cassava, pastures, and sugarcane [4, 26–29]. It is believed that one preponderant factor in the evolution of its digestive physiology would be the possibility of heterogeneous diets. The type and levels of peptidases produced are probably adjusted by the types of food consumed by the insect. While many authors highlight the diet as the main factor in adaptive patterns, the importance of inheritance of ancestral characteristics is undeniable in most cases [30].

### Glycosyl hydrolases in *M*. *fryanus* larvae

**Fig 7:**
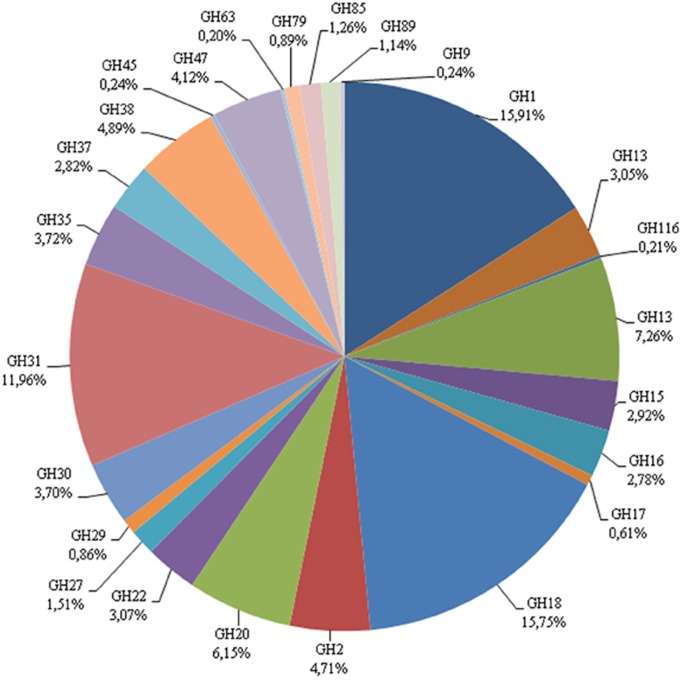
Glycosyl Hydrolase families found in gut transcriptomes of *M*. *fryanus* larvae. GH18 (Chitinase) is the most abundant, followed by GH2 (β-glucuronidase), GH1 (β-glucosidase), and GH31 (α-glucosidase).

*M*. *fryanus* gut (sum of foregut, midgut, and hindgut) transcriptomes revealed a large number of enzymes related to carbohydrate metabolism. Glycosyl hydrolases (GHs) involved in plant cell wall degradation have been less well studied as their phylogeny is too complex. Moreover, GHs also have potential applicability in biotechnology industry [31]. To determine the distribution of GHs involved in *M*. *fryanus* biology, CAZY database was used for the annotation of these enzymes. The distribution graph (Fig 7) shows a large number of GH families in gut transcriptomes, such as GH1, GH9, GH16, GH27, GH35, GH38, GH31, GH45, and GH47. From a total of 458 unigenes, the most abundant were GH1, GH31, and GH18, while the less abundant were GH9, GH45, and GH63, represented as singletons.

#### Hemicellulose degradation

The metabolism of hemicellulose is attributed to xylanases and enzymes that act in minor polysaccharides, such as β-xylosidases, β/α-galactosidases, and α-mannosidases. Though endogenous xylose utilization capabilities have not been described in cerambycids, xylanases from *Anoplophora glabripennis* genome have been described. These sequences have high identity with *Tribolium castaneum* β-glucosidase [9,32] *A*. *glabripennis* xylanase activity was confirmed by zymography of a native protein [32]. So, despite β-glucosidase identity, these *A*. *glabripennis* sequences act as xylanases. All annotated *M*. *fryanus* GH1 sequences found in these transcriptomes were analyzed, and they showed sequence similarity with β-glucosidase from GenBank (NCBI). This suggests that *M*. *fryanus* GH1 sequences can act as xylanases similar to *A*. *glabripennis* ones.

The presence of annotated GH35 β-galactosidases, GH38 α-mannosidases, and GH47 α-mannosidases in *M*. *fryanus* transcriptomes indicates that hemicellulose degradation can reach the monomerization stage. This underscores the putative capability of *M*. *fryanus* to use hemicellulose as an energy source, as previously suggested in *Diabrotica virgifera virgifera* [17].

#### Cellulose degradation

Cellulose degradation is widely studied in insect biology. Some coleopteran species are capable of digesting cellulose by their own arsenal of cellulases and are not dependent on enzymes from microorganisms [9,17,31]. The gut transcriptomes of *M*. *fryanus* revealed two different cellulases, GH45 (*Mf_GH45*) and GH9 (*Mf_GH9*). Coleopteran GH45 enzymes have a peculiarity in the proton donor residue, which can be aspartate, found specifically in Curculionidae, or glutamate [17,31]. *Mf_GH45* has Glu as proton donor amino acid, similar to other Cerambycidae GH45 sequences [31]. In Coleoptera, GH45 genes are found in Chrysomeloidea superfamily and Curculionidae family [17,31], but not in other families with distinct ancestry. This fact suggests that this enzyme was acquired by horizontal gene transfer from microorganisms through a common ancestor or by means of convergent evolution with GH9 since they have similar binding domains [33].

**Fig 8:**
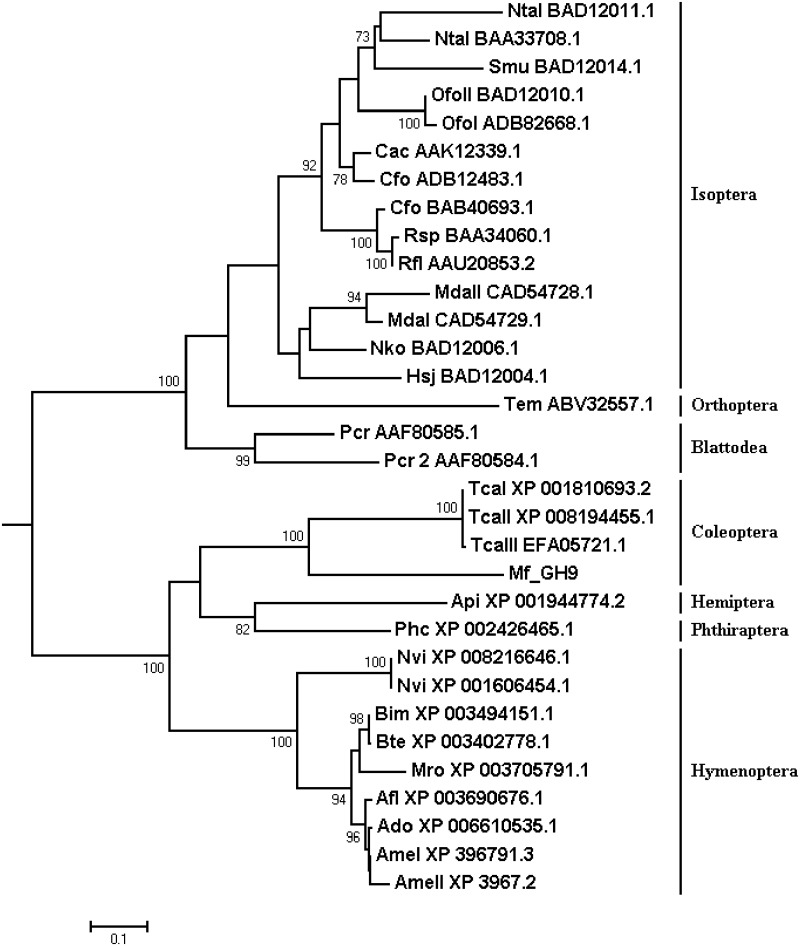
Phylogenetic tree based on glycosyl hydrolases from insect family 9. Alignment was performed with MAFFT v7.031b using the L-INS-I algorithm with the–maxiterate option set to 1000, determined using ProtTest. Phylogenetic reconstruction was carried out with RaxML v7.2.7. Bootstrap values were achieved after 1,000 pseudo replicates, indicated in branches. All the sequences, except *Mf*_GH9, are represented by their compressed names followed by NCBI access numbers.

GH9 was never found in any insect belonging to the Chrysomeloidea superfamily or Curculionidae family [17]. However, *M*. *fryanus* transcriptome showed an unigene corresponding to GH9 family. The alignment of *Mf_GH9* with *T*. *castaneum* GH9 (TcEG1) sequence [34] resulted in a high similarity profile. Thus, *Mf_GH9* and TcEG1 share a close ancestry as confirmed by the phylogenetic tree (Fig 8). *T*. *castaneum* sequences formed a specific branch, segregated by a bootstrap node support of 100 from other orders. However, the phylogenetic tree showed that coleopteran GH9, although weakly represented, evolved differently into the order, since *Mf_GH9* and TcEG1 were not in the same branch. More sequences from more beetles need to be investigated to elucidate the evolutionary history of GH9 in Coleoptera.

GH16 family genes are largely found in insects, but not all genes encode enzymes with β-1,3-glucanase activity [17]. Duplication copy event of GH16 family genes originated a group of pattern recognition proteins called Gram-negative bacteria-binding proteins (GNBPs) or β-1,3-glucan recognition proteins [31,35,36], and they are involved in innate immune recognition [37–40]. The β-1,3-glucanase active site is not conserved in GNBPs, and the automatic annotation categorizes them as GH16 because of the conserved β-1,3-glucan-binding domain in their N-terminal region [31,35,40–42]. *M*. *fryanus* gut transcriptomes revealed two β-1,3-glucanase-like (*Mf_1*,*3gl1* and *Mf_1*,*3gl2*) and two GNBP-like (*Mf_GNBP1* and *Mf_GNBP2*) sequences. Probably *Mf_1*,*3gl1* and *Mf_1*,*3gl2* act in callose degradation as this tissue is composed of β-1,3 linkages [43]. *Mf_GNBP1* and *Mf_GNBP2* probably act on *M*. *fryanus* immune system, as seen in other beetles like *T*. *castaneum*, *Tenebrio molitor*, *Dendroctonus ponderosae*, and *Diabrotica virgifera virgifera* [17].

#### Expression patterns of GH transcripts from *M*. *fryanus*

This transcriptome analysis showed glycosyl hydrolase sequences from different families. Five of them were chosen to verify their expression profile in different larval parts. They were GH13 annotated as α-amylase, GH35 as β-galactosidase, GH116 as β-glucosylceramidase, GH16 as a putative GNBP protein, and GH9 as endoglucanase.

GH13 (Fig 6F) and GH35 (Fig 6G) showed the highest expression in the midgut, but not in other parts of the *M*. *fryanus* larvae body. FPKM values for GH13 and GH35 reinforce the highest expression in midgut. This profile is expected since most of the digestive enzymes are expressed mainly in the midgut of other insects belonging to the same family [44–46]. The presence of α-amylase and β-galactosidase enzymes in the *M*. *fryanus* digestive system suggests that starch and hemicellulose from sugarcane are carbon sources the insect feed on.

GH116 showed the highest expression in the midgut and a basal expression in other parts of the *M*. *fryanus* larvae (Fig 6H). FPKM for GH116 showed a higher expression in gut tissues, mainly in midgut, corroborating the qPCR results. The presence of glucosylceramidase in *A*. *glabripennis* was related to modulating the interactions with symbiotic microorganisms associated with the midgut [9]. Thus, GH116 from *M*. *fryanus* can be a non-lysosomal enzyme acting on the regulation of symbiotic interactions.

The insect’s innate immune system is based on recognition molecules and antimicrobial peptides. They are produced mainly in fat body, relative of the liver, and in the epithelium, such as digestive tracts and integument [47]. qPCR tests for GH16 showed a higher expression in digestive tracts, integument, head, and fat body (Fig 6I). These results show that this gene is one among the *Mf_GNBP*s, and the protein encoded by it acts as a recognition molecule from innate immune system. On the contrary, FPKM results did not show a high expression level in fat body, and the tissue with a higher expression was the midgut. CAZY database correlates GH16 with enzymes active against xylan and glucan substrates, and the highest expression in the midgut could explain a digestive function of this protein. Therefore, activity tests are necessary to better understand the actual role of this protein.

Interestingly, GH9 analysis showed a different pattern of expression than digestive enzymes. There was a higher expression in head, followed by foregut and hindgut (Fig 6J). The highest expression in head can be explained by the presence of salivary glands. They contribute to digestion and are located in the larval head. Probably, GH9 enzymes are secreted by the salivary glands to help cellulose disruption, the first step in digestion. A low expression in the foregut and a negligible expression in the midgut suggest that this tissue acts like a reactor whereby this enzyme could liberate its products allied to its absorption. Surprisingly, it was observed in the expression patterns related to the hindgut. In general, the digestive enzymes are not produced in the insect’s hindgut [53], but another study reported the expression of an endoglucanase GHF45, a cellulase, in the hindgut of *Diabrotica virgifera virgifera* [48].

### Serpins in *M*. *fryanus* transcriptomes

Serpins (SERine Protease INhibitors) are a superfamily of protease inhibitors involved in many biological processes. They are found in a large number of organisms, including plants, animals, viruses, but rarely in fungi, bacteria, and archaea [49]. They are structurally heterogeneous with an average size of 350–400 residues and molecular weight ranging between 40 kDa and 50 kDa. Serpins form an irreversible complex between their reactive center loop situated near the C-terminus and the action site of the target protease leading to the inhibition of proteolysis [50].

**Fig 9:**
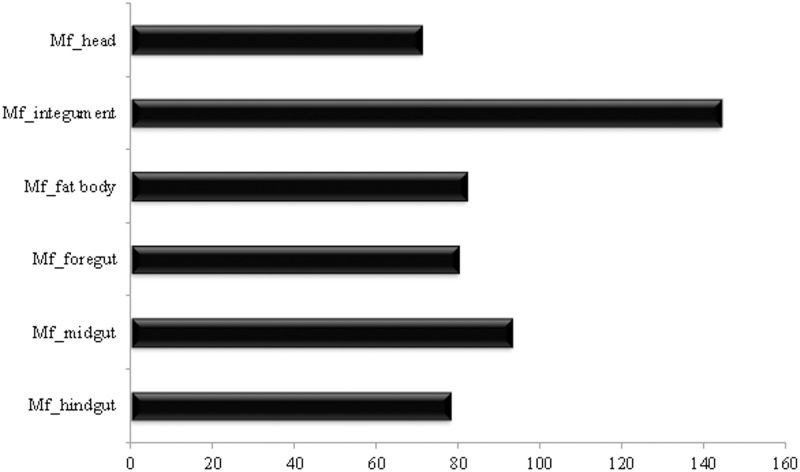
Relative abundance of similar serpin sequences in different parts of *M*. *fryanus*. Normalization was accomplished by multiplying the number of sequences obtained for each serpin by the number of transcripts from each tissue (see Table 3) divided by the total number of transcripts generated (78,498).

In insects, serpins are described in the immune system, modulation of hemolymph coagulation, prophenoloxidase activation, and induction of synthesis of antimicrobial peptides [51]. Some studies have shown efficiency in the use of serpins for insect control [45,52–55]. The use of RNAi to silence serpins for pest control has been reported previously [8,45,56–60]. After the annotation using PFAM database, we searched for serpins using the Mf_all database. We identified 89 transcripts as serpins and found that 25 had complete ORFs. We selected one representative ORF to serpin family to be aligned with the transcripts of the local database of each part (head, integument, fat body, and guts) using Blastp algorithm with a cut-off value of 1.0e^-5^. After this, we counted the number of different contigs presenting each protein family. There was an abundance of serpins in integument, followed by midgut, fat body, foregut, hindgut, and head (Fig 9).

Serpins are abundant in insect integument [61]. It is known that cuticle and midgut act as barriers against invading pathogens. Insect peptidases and their inhibitors play an important role in the innate immune response. Furthermore, they have been found to be involved in cuticular protein degradation [61–63]. Serpin expression is lowered during instar changing, but subsequently increases in order to suppress the action of serine peptidases present in integument, allowing the production of a new cuticle [62]. However, it is known that the cuticle prophenoloxidase system is important for melanization processes and defense reactions [64,65].

#### Expression analysis of serpin genes in *M*. *fryanus*

Tissue-specific expression of three serpin genes was analyzed for *M*. *fryanus* transcriptomes. Although these serpins are very similar in their protein sequence (differ only in the C-terminus portion, where the inhibition loop is located), this study showed that these serpins have differential expression levels (Fig 6). The selected serpins were labeled Mf_S1, Mf_S2, and Mf_S3. By both FPKM and qPCR alike, Mf_S1 (Fig 6K) was expressed more in fat body, foregut, and hindgut. Mf_S2 (Fig 6L) presented a ubiquitous expression with a maximum expression in fat body, and Mf_S3 (Fig 6M) was mainly expressed in the foregut.

The higher serpin expression in fat body, mainly Mf_S1, is probably because this is one of the main sites of synthesis of proteins related to innate immunity. Mf_S2 showed a relative ubiquitous expression. This expression pattern across larval parts indicates that this putative serpin has pleiotropic effects [61]. The insect’s intestinal tract can be classified as a tissue primarily involved in digestion and detoxification. However, studies have revealed that a great diversity of genes are involved in innate immunity throughout the insect’s intestinal tract, in the form of GNBPs, peptidoglycan recognition proteins, lysozymes, serpins, and others. This justifies the fact that we found high higher expression levels of Mf_S3 in intestinal tissues (mainly in the foregut). Serpins produced in the intestinal tract could have important roles in the insect’s immune defense, including the control of digestive peptidases present in the gut.

### Unknown ORFs and potential targets

**Fig 10:**
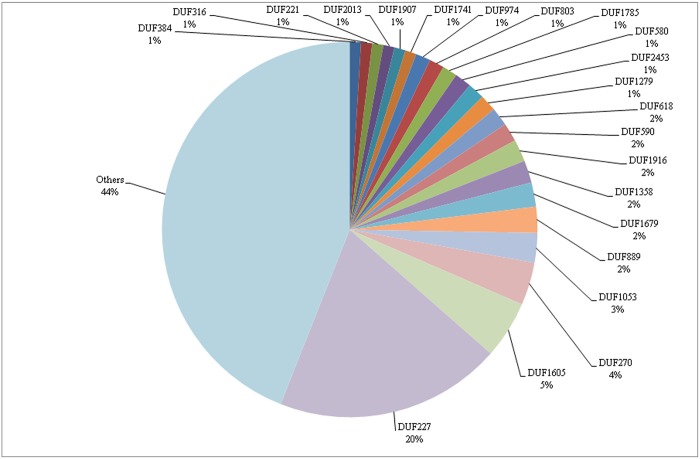
Distribution of the 1% of contigs classified as unknown. DUF was attributed as available in the PFAM system.

The unknown ORFs have potential biotechnological applications but still remain undescribed in many datasets, as also ours. *M*. *fryanus* accounts for about 1% of unknown contigs in the sum of all transcriptomes (Fig 10). Several putative families of domain of unknown function (DUF) were observed in our datasets during annotation using PFAM database.

DUF227 represents almost 20% of all unknown reads, and it was recently reclassified as EcKinase (PF02958). This family includes enzymes like ecdysteroid 22-kinase, responsible for the phosphorylation of ecdysteroids to physiologically inactive ecdysteroid 22-phosphates, and this process is linked to insect growth and metabolism of moulting hormones [66]. This class of ORFs could reveal the processes of ecdysis and metamorphosis of this insect, bringing a better understanding of the beetle’s life cycle.

Otopetrin family (PF03189), previously assigned as DUF270, comprised 4% of the unknown contigs. These proteins are restricted to metazoa, forming transmembrane structures [67] and modulating calcium homeostasis and influx of calcium in response to extracellular ATP [68]. They act in the otoconia/otoliths formation, which works like sensors for gravity and acceleration. They are required for the processing of information regarding spatial orientation and acceleration [68–70]. Due to the fossorial habits of *M*. *fryanus*, investigations concerning this otopetrin-like gene family and its putative whole involving spatial orientation through underground tunnels could reveal another interesting molecular target to its control.

A preliminary FPKM analysis showed that otopetrin was expressed only in fat body, reinforcing the idea that otoconia/otoliths act like acceleration sensors. However, our qPCR analysis (Fig 6N) revealed a higher expression of otopetrin-like mRNAs in fat body and integument, followed by head, and a lower expression in guts. These preliminary results suggest fat body and integument are environments that favor the development of otolyths/otoconia, which may possible contitute gravity-sensing organs in *M*. *fryanus*. Otopetrin-like genes were previously reported in transcriptomes of several arthropods, but were not referred like this, until very recently when protein family DUF270 was reassigned. In coleopterans, only *T*. *castaneum* has been assigned with otopetrin-like ESTs [67], although it was not further explored or analyzed.

Otopetrin overexpression can modulate purinergic-mediated Ca^2+^ homeostasis [68], and is correlated with otoliths formation ratio. In this way, this gravity sensor will be increased in soft tissues, where otoliths could generate pressure through increasing acceleration or gravity to indicate the relative positions of insect/larvae. Fat body and integument are interesting since they cover the whole body and can serve in sensor purposes. The expression ratios of otopetrin-like mRNAs observed in this study is the first experimental prove of its presence in beetles and reinforces the hypothesis that this insect can survive underground for a long time during early developmental stages, and otopetrin can be related with their subterranean orientation. This protein seems to be a promising molecular target for *M*. *fryanus* control, since a silencing of this gene could hamper the larvae from emerging to the surface to feed on plant rhizome and reproduce and it may help us to understand why these larvae pupped only at great depths.

Each of the following motifs represented about 2% in the final amount: DUF618, DUF590, DUF1916, DUF1358, DUF1679, and DUF889. They may encode some interesting molecules and are not yet validated. Remarkably, DUF590 is believed to be an anoctamin (PF04547), a family that does not present any similarity to other known channel proteins, being a calcium-activated ionic channel [71]. This DUF is expressed in various secretory epithelia (like retina and sensory neurons) and mediates receptor-activated chloride currents in diverse physiological processes [72].

## Conclusions

By combining Illumina HiSeq platform for NGS and RNA-Seq method, we sequenced, assembled, annotated, and made public *de novo* transcriptomes of sugarcane rhizome borer, *M*. *fryanus*. These transcriptomes represent the first genetic study about this insect pest, with a total of 53,529 protein sequences being annotated. Data obtained from this study will contribute to a significant understanding of the biology of this important pest.

From our data, we drew more interesting information regarding the digestive physiology of *M fryanus*. The presence of cysteine and serine peptidases along the digestive tract seems to favor this insect in exploring different diet possibilities. Selected GHs appear to be related to digestive (cellulolytic degradation) and immunological (recognition molecules and antimicrobial peptides) functions. Serpins might be involved in many biological processes. In *M*. *fryanus*, it appears to be active mainly in immune system and for control of digestive action. We identified an otopetrin-like transcript being expressed in fat body. Probably otocolyth formation is expected to play a role in gravity sensing. Besides, to the best of our knowledge, the present report is the first regarding differential expression of this gene in a Coleoptera. The otopetrin-like transcript seems to be a promising target for pest control, since a silencing of this gene could hamper the larvae from emerging to the surface to feed or reproduce.

The *M*. *fryanus* transcriptomic data generated in this study can help accelerate future studies to understand the diverse aspects of the insect physiology and biochemistry.

## Supporting information

S1 Table Nucleotide and aminoacids sequences of the ORFs used in this work. (XLSX)

S2 Table Validation of ribosomal protein RP18 as an internal control for qPCR. The Friedman test revealed no statistical variation between the means of Ct values observed for the different parts of larval body.
(XLSX)
